# Supplementary material for: Social aging trajectories are sex-specific, sensitive to adolescent stress, and most robustly revealed during social tests with familiar stimuli
Source: bioRxiv. 2023 Apr 28:2023.04.27.538622. Preprint. [Version 1] doi: 10.1101/2023.04.27.538622 (PMC10168396; doi:10.1101/2023.04.27.538622)
Supplement: 1 [file NIHPP2023.04.27.538622V1-supplement-1.pdf]

bioRxiv preprint doi: https://doi.org/10.1101/2023.04.27.538622; this version posted April 28, 2023. The copyright holder for this preprint (which was not certified by peer review) is the author/funder, who has granted bioRxiv a license to display the preprint in perpetuity. It is made available under aCC-BY 4.0 International license.

**Supp. Fig. 1**

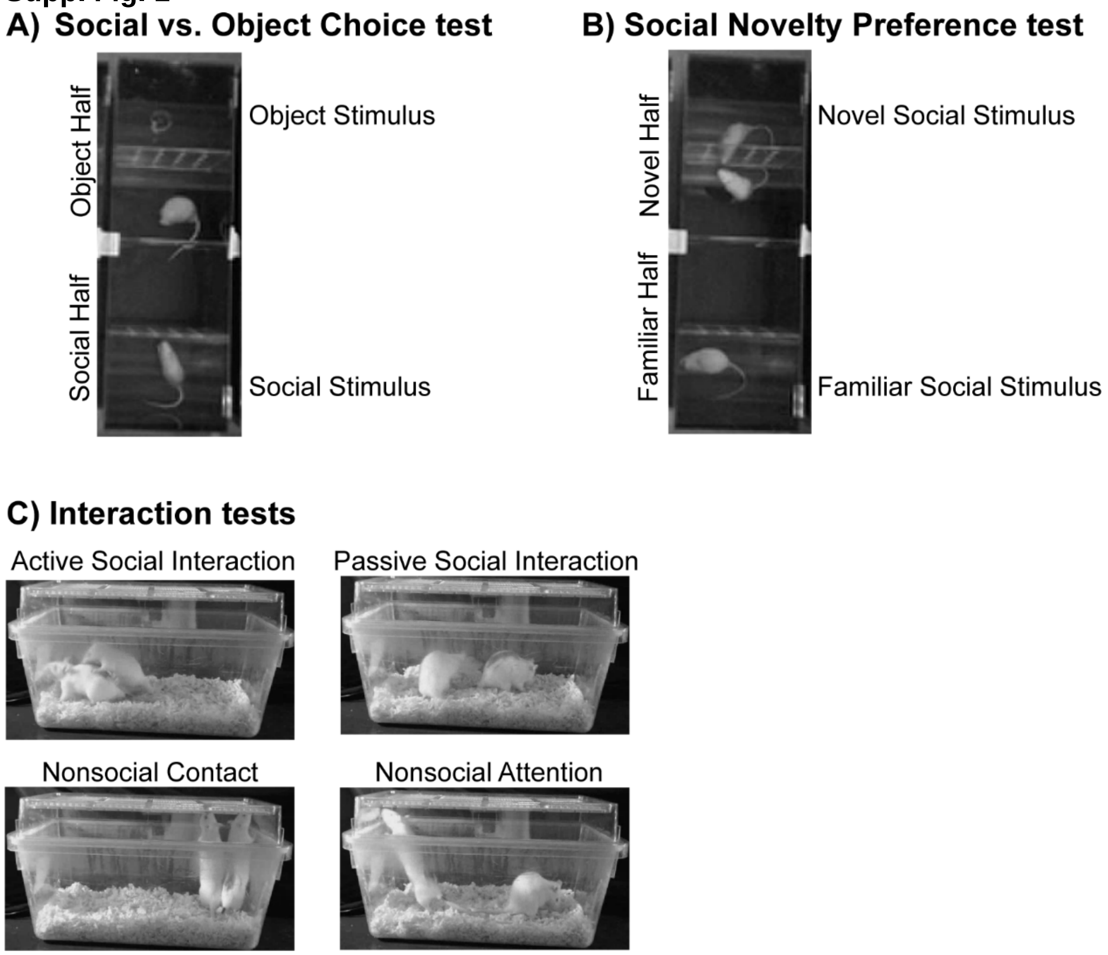


**Supp. Fig. 1: Representative images of social tests**
Depiction of social choice tests **(A-B)** and behavioral quantification of Interaction tests **(C)**.

bioRxiv preprint doi: https://doi.org/10.1101/2023.04.27.538622; this version posted April 28, 2023. The copyright holder for this preprint (which was not certified by peer review) is the author/funder, who has granted bioRxiv a license to display the preprint in perpetuity. It is made available under aCC-BY 4.0 International license.

**Supp. Fig. 2**

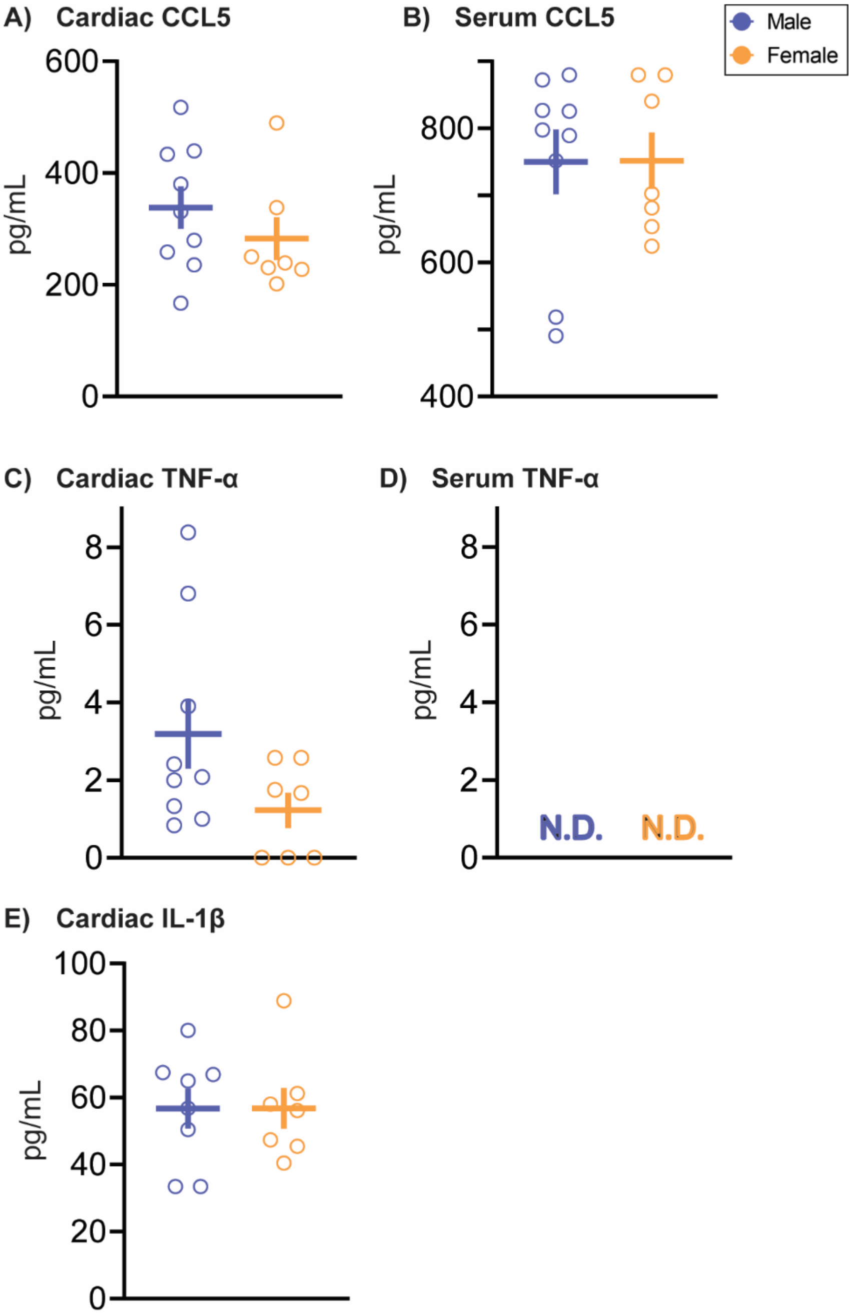


**Supp. Fig. 2: No sex differences are detected in naturally aged rats in protein expression of immune chemokines and cytokines CCL5, TNF-α, or IL-1β in heart and serum**
At termination of all studies (~13mo), rats were euthanized and heart and serum collected for ELISA analysis. There was no statistically significant sex difference in **(A-B)** CCL5 protein expression in heart or serum, **(C)** TNF-α expression in heart, or **(E)** IL-1β expression in heart. **(D)** There was no detectable (N.D.) TNF-α expression in serum in either sex. Protein concentration loaded for ELISAs was normalized using prior BCA analysis. Histograms depict average ± SEM. *n*=7-9 rats/sex/group.

bioRxiv preprint doi: https://doi.org/10.1101/2023.04.27.538622; this version posted April 28, 2023. The copyright holder for this preprint (which was not certified by peer review) is the author/funder, who has granted bioRxiv a license to display the preprint in perpetuity. It is made available under aCC-BY 4.0 International license.

**Supp. Fig. 3**

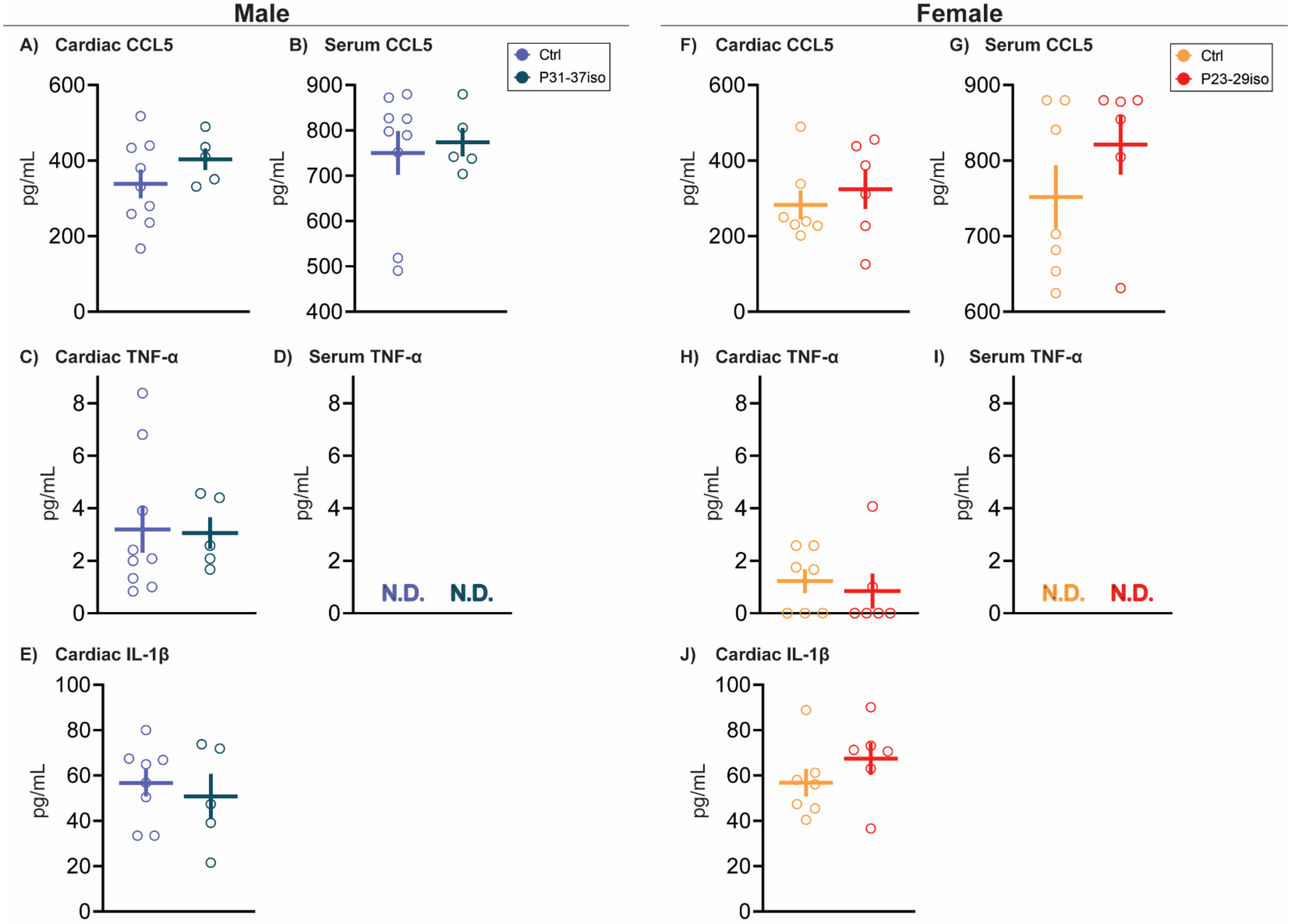


**Supp. Fig. 3: Social isolation during sex-specific adolescent critical periods does not impact protein expression of immune chemokines and cytokines CCL5, TNF-α, or IL-1β in heart and serum**
Rats are single-housed during sex-specific critical periods for adolescent social development, P31-37 in males and P23-29 in females, and then re-housed with previous cage mates. At termination of all studies (~13mo), rats were euthanized and heart and serum collected for ELISA analysis. There was no statistically significant effect of adolescent manipulation in either sex in **(A-B, F-G)** CCL5 protein expression in heart or serum, **(C, H)** TNF-α expression in heart, or **(E, J)** IL-1β expression in heart. **(D, I)** There was no detectable (N.D.) TNF-α expression in serum in either sex. Protein concentration loaded for ELISAs was normalized using prior BCA analysis. Histograms depict average ± SEM. *n*=5-9 rats/sex/group.
